# The antitumour activities of statins

**DOI:** 10.3747/co.2007.159

**Published:** 2007-12

**Authors:** H.K. Takahashi, M. Nishibori

Abnormally elevated levels of serum cholesterol have been demonstrated to contribute to atherosclerosis and coronary artery disease. Statins, inhibitors of 3-hy-droxy-3-methylglutaryl coenzyme A (hmg-Coa) re-ductase, are efficient and widely used drugs in the treatment of lipid disorders, especially hypercholes-terolemia. In addition to their cholesterol-lowering effects, statins are reported to inhibit tumour cell growth 1,2. Statins are also known to synergistically enhance the effects of chemotherapy 3,4 and to overcome chemoresistance 5. Accordingly, statins prolong the survival of patients with hepatocellular carcinoma^4^, and they reduce the risk of colorectal cancer^6^ and breast cancer^7^.

Statins induce apoptosis and reduce cell invasiveness in various cell lines, including malignant glioma^8^, neuroblastoma^9^, myeloid leukemia^10^, and breast carcinoma^11^. Cancer cells overexpress hmg coa reductase 12. The chemopreventive activity of statins against cancer is suggested to depend on inhibition of hmg-Coa reductase in cholesterol synthesis and, thereby, cell growth 13. The Ras protein is important in the regulation of cell differentiation and proliferation. Statins are reported to inhibit the activation of *ras* 14. The products of the mevalonate pathway are necessary for diverse cellular functions, including the G1–S phase transition of cell proliferation and the formation of cell membranes 15. Statins may therefore inhibit cancer cell growth and lead to apoptotic cell death through their inhibition of the mevalonate pathway, although other mechanisms also have been suggested.

Interleukin-18 (il-18), a monocyte-derived cyto-kine, is upstream of the production of interferon γ from T cells and natural killer cells 16,17. Interleukin-18 is known to play an important role in regulating immune responses, exhibiting significant antitumour activity 18. The antitumour effects of il-18 are mediated by activation of natural killer cells and cytotoxic T lymphocytes 19. In a previous study, we found that the statins pravastatin, fluvastatin, and simvastatin induced production of il-18 by human monocytes 20,21. The effects of pravastatin, fluvastatin, and simvastatin were abolished by the addition of mevalonate, indicating the involvement of hmg-Coa reductase in the action of the tested statins.

Angiogenesis is characterized by the formation of new capillaries from existing vessels. It is well known that tumour growth and metastasis both require growth of new blood vessels 22,23. The statins lovastatin and cerivastatin are reported to inhibit tumour-induced angiogenesis by reducing metabolites of the mevalonate pathway that are pivotal in angio-genesis 24,25.

The foregoing observations suggest that the an-ticancer effect of statins depends on the apoptosis of cancer cells, the production of il-18 by monocytes, and the inhibition of angiogenesis. However, the effects of statins on cancer are not completely understood. Further experimental research will be useful in clarifying this complex relationship.

## References

[1] Wong WW, Tan MM, Xia Z, Dimitroulakos J, Minden MD, Penn LZ (2001). Cerivastatin triggers tumor-specific apoptosis with higher efficacy than lovastatin. Clin Cancer Res.

[2] Paragh G, Kertai P, Kovacs P, Paragh G, Fülöp P, Foris G (2003). hmg coa reductase inhibitor fluvastatin arrests the development of implanted hepatocarcinoma in rats. Anticancer Res.

[3] Li HY, Appelbaum FR, Willman CL, Zager RA, Banker DE (2003). Cholesterol-modulating agents kill acute myeloid leukemia cells and sensitize them to therapeutics by blocking adaptive cholesterol responses. Blood.

[4] Holstein SA, Hohl RJ (2001). Synergistic interaction of lovastatin and paclitaxel in human cancer cells. Mol Cancer Ther.

[5] Bogman K, Peyer AK, Török M, Küsters E, Drewe J (2001). hmg-coa reductase inhibitors and P-glycoprotein modulation. Br J Pharmacol.

[6] Poynter JN, Gruber SB, Higgins PD (2005). Statins and the risk of colorectal cancer. N Engl J Med.

[7] Boudreau DM, Gardner JS, Malone KE, Heckbert SR, Blough DK, Daling JR (2004). The association between 3-hydroxy-3-methyl-glutaryl coenzyme A inhibitor use and breast carcinoma risk among postmenopausal women: a case-control study. Cancer.

[8] Jones KD, Couldwell WT, Hinton DR (1994). Lovastatin induces growth inhibition and apoptosis in human malignant glioma cells. Biochem Biophys Res Commun.

[9] Dimitroulakos J, Yeger H (1996). hmg-coa reductase mediates the biological effects of retinoic acid on human neuroblastoma cells: lovastatin specifically targets P-glycoprotein–expressing cells. Nat Med.

[10] Clutterbuck RD, Millar BC, Powles RL (1998). Inhibitory effect of simvastatin on the proliferation of human myeloid leukaemia cells in severe combined immunodeficient (scid) mice. Br J Haematol.

[11] Kotamraju S, Willams CL, Kalyanaraman B (2007). Statin-induced breast cancer cell death: role of inducible nitric oxide and ar-ginase-dependent pathways. Cancer Res.

[12] Hentosh P, Yuh SH, Elson CE, Peffley DM (2001). Sterol-independent regulation of 3-hydroxy-3-methylglutaryl coenzyme A reductase in tumor cells. Mol Carcinog.

[13] Buchwald H (1992). Cholesterol inhibition, cancer, and chemotherapy. Lancet.

[14] Goldstein JL, Brown MS (1990). Regulation of the mevalonate pathway. Nature.

[15] Wong WW, Dimitroulakos J, Minden MD, Penn LZ (2002). hmg-coa reductase inhibitors and the malignant cell: the statin family of drugs as triggers of tumor-specific apoptosis. Leukemia.

[16] Okamura H, Tsutsi H, Komatsu T (1995). Cloning of a new cytokine that induces ifn-γ production by T cells. Nature.

[17] Ahn HJ, Maruo S, Tomura M (1997). A mechanism underlying synergy between il-12 and ifn-γ–inducing factor in enhanced production of ifn-γ. J Immunol.

[18] Osaki T, Hashimoto W, Gambotto A (1999). Potent antitumor effects mediated by local expression of the mature form of the interferon-γ inducing factor, interleukin-18 (il-18). Gene Ther.

[19] Micallef MJ, Tanimoto T, Kohno K, Ikeda M, Kurimoto M (1997). Interleukin 18 induces the sequential activation of natural killer cells and cytotoxic T lymphocytes to protect syngeneic mice from transplantation with Meth A sarcoma. Cancer Res.

[20] Takahashi HK, Mori S, Iwagaki H (2005). Differential effect of LFA703, pravastatin, and fluvastatin on production of il-18 and expression of icam-1 and cd40 in human monocytes. J Leukoc Biol.

[21] Takahashi HK, Mori S, Iwagaki H, Yoshino T, Tanaka N, Nishibori M (2005). Simvastatin induces interleukin-18 production in human peripheral blood mononuclear cells. Clin Immunol.

[22] Saaristo A, Karpanen T, Alitalo K (2000). Mechanisms of angiogen-esis and their use in the inhibition of tumor growth and metastasis. Oncogene.

[23] Hanahan D, Folkman J (1996). Patterns and emerging mechanisms of the angiogenic switch during tumorigenesis. Cell.

[24] Vincent L, Chen W, Hong L (2001). Inhibition of endothelial cell migration by cerivastatin, an hmg-coa reductase inhibitor: contribution to its anti-angiogenic effect. FEBS Lett.

[25] Feleszko W, Balkowiec EZ, Sieberth E (1999). Lovastatin and tumor necrosis factor-α exhibit potentiated antitumor effects against Ha-*ras-*transformed murine tumor via inhibition of tumor-induced angiogenesis. Int J Cancer.

